# 

**Published:** 1897-05-29

**Authors:** 


					OADBURY'S
, COCOA
" The standard of highest purity at present
attainable,"?Lancet.
CADBURY'S 0
COCOA
NO ALKALIES USED.
WHICH HOSPITAL
No.^oi Vol. XXII. Saitoday,
May 29th, 1897.
[SubscriptionTerms,] pRICE TWOPENCE.

				

